# Anatomical substrates and connectivity for bradykinesia motor features in Parkinson’s disease after subthalamic nucleus deep brain stimulation

**DOI:** 10.1093/braincomms/fcad337

**Published:** 2023-12-13

**Authors:** Min Jae Kim, Yiwen Shi, Jasmine Lee, Yousef Salimpour, William S Anderson, Kelly A Mills

**Affiliations:** 1 Movement Disorders Division, Department of Neurology, Johns Hopkins School of Medicine, 600 N. Wolfe Street, Baltimore, MD 21287, USA; Department of Neurosurgery, Johns Hopkins School of Medicine, 600 N. Wolfe Street, Baltimore, MD 21287, USA; Department of Biomedical Engineering, Johns Hopkins University, 3400 N. Charles St, Baltimore, MD 21218, USA; 1 Movement Disorders Division, Department of Neurology, Johns Hopkins School of Medicine, 600 N. Wolfe Street, Baltimore, MD 21287, USA; 1 Movement Disorders Division, Department of Neurology, Johns Hopkins School of Medicine, 600 N. Wolfe Street, Baltimore, MD 21287, USA; Department of Neurosurgery, Johns Hopkins School of Medicine, 600 N. Wolfe Street, Baltimore, MD 21287, USA; Department of Neurosurgery, Johns Hopkins School of Medicine, 600 N. Wolfe Street, Baltimore, MD 21287, USA; Department of Biomedical Engineering, Johns Hopkins University, 3400 N. Charles St, Baltimore, MD 21218, USA; 1 Movement Disorders Division, Department of Neurology, Johns Hopkins School of Medicine, 600 N. Wolfe Street, Baltimore, MD 21287, USA

**Keywords:** Parkinson’s disease, deep brain stimulation, bradykinesia, subthalamic nucleus

## Abstract

Parkinsonian bradykinesia is rated using a composite scale incorporating the slowed frequency of repetitive movements, decrement amplitude and arrhythmicity. Differential localization of these movement components within the basal ganglia will drive the development of more personalized network-targeted symptomatic therapies. In this study, using an optical motion sensor, we evaluated the amplitude and frequency of hand movements during a grasping task with subthalamic nucleus deep brain stimulation ‘on’ or ‘off’ in 15 patients with Parkinson’s disease. The severity of bradykinesia was assessed blindly using the Unified Parkinson’s Disease Rating Part III scale. The volumes of activated tissue of each subject were estimated where changes in amplitude and frequency were mapped to identify distinct anatomical substrates of each component in the subthalamic nucleus. The volumes of activated tissue were used to seed a normative functional connectome to generate connectivity maps associated with amplitude and frequency changes. Deep brain stimulation–induced change in amplitude was negatively correlated with a change in Unified Parkinson’s Disease Rating Part III scale for right (*r* = −0.65, *P* < 0.05) and left hand grasping scores (*r* = −0.63, *P* < 0.05). The change in frequency was negatively correlated with amplitude for both right (*r* = −0.63, *P* < 0.05) and left hands (*r* = −0.57, *P* < 0.05). The amplitude and frequency changes were represented as a spatial gradient with overlapping and non-overlapping regions spanning the anteromedial–posterolateral axis of the subthalamic nucleus. Whole-brain correlation maps between functional connectivity and motor changes were also inverted between amplitude and frequency changes. Deep brain stimulation–associated changes in frequency and amplitude were topographically and distinctly represented both locally in the subthalamic nucleus and in whole-brain functional connectivity.

## Teaser

Kim *et al*. report on subthalamic nucleus deep brain stimulation–induced volumes of tissue activation associated with individual components of Parkinson’s disease bradykinesia, showing separate sites associated with the amplitude and frequency of hand movements. They also show that the stimulation of the amplitude ‘sweetspot’ correlates most strongly with changes in motor rating scales.

## Graphical Abstract

**Graphical Abstract:**
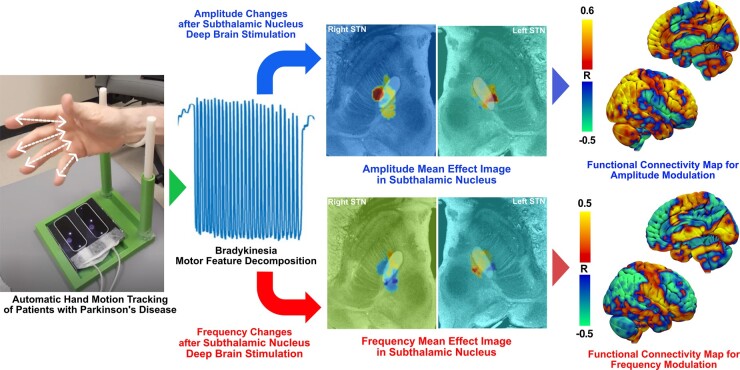

## Introduction

Deep brain stimulation (DBS) is a well-established treatment for refractory motor complications of Parkinson’s disease, and most often targets the subthalamic nucleus (STN) or internal pallidum.^1,2^ STN-DBS is the most common procedure internationally, and is used to target tremor, rigidity and bradykinesia.

To assess the baseline severity of bradykinesia and its clinical changes after therapeutic interventions, the Movement Disorders Society-Unified Parkinson’s Disease Rating scale (MDS-UPDRS III) is widely adopted in clinics since it provides a reliable quantitative measure of bradykinesia severity through different tasks in both upper and lower extremities.^3^ However, bradykinesia ratings represent a composite score encompassing multiple motor features. For example, a single rating, ranging from 0 to 4, can be determined in the presence of loss of rhythm or interruptions, slowing or amplitude decrements during the task.^3^ As such, potentially independent motor features may differentially influence the assessment of bradykinesia based on the MDS-UPDRS III rating.^4,5^ At the individual level, people with Parkinson’s disease may experience varying degrees of hypokinesia, bradykinesia (pure slowing) or sequencing effect, and this heterogeneity may complicate the development of circuit-based therapeutic to optimize the treatment of ‘bradykinesia’ at the individual level. In fact, at a group level, bradykinesia has a smaller degree of improvement than that of tremor or rigidity.^6^

Recent neuroimaging studies have highlighted the presence of spatially localized ‘sweet spots’ in the STN for DBS, the stimulation of which may result in superior motor improvement.^7-9^ However, neural substrates within the STN associated with changes in individual bradykinesia components are not well characterized. Understanding the neural substrates representing independent motor features of bradykinesia would help elucidate the larger role that the STN plays in complex human motor function. Furthermore, DBS provides a unique opportunity to study the systems neuroscience of motor control by deconstructing parallel circuitry through the STN by stimulating specific sub-regions. This approach would help describe the representation of motor features of bradykinesia both within the basal ganglia and across larger cortical networks. Clinically, understanding a refined ‘sweet spot’ for each of these motor features could aid in tailoring imaging-based DBS programming to specifically target a given patient’s symptoms and signs. It could also potentially help develop more personalized DBS therapy approaches that target the most prominent components of bradykinesia in an individual patient.

In this study, we first investigated changes in two separate motor features—amplitude and frequency—and their association with the overall MDS-UPDRS III bradykinesia rating after STN-DBS was applied. Based on these findings, we then explored how these changes in motor features were represented in both local STN stimulation sites and larger cortical network levels through functional connectivity (FC) analysis.

## Materials and methods

### Patient recruitment

Patients diagnosed with clinically established Parkinson’s disease^10^ who underwent bilateral STN-DBS with stable stimulation parameters for at least 3 months at the Johns Hopkins Neuromodulation and Advanced Therapies Clinic were recruited for the study (internal IRB number: IRB00270213). Patients are evaluated for advanced therapies with a multidisciplinary approach that includes medication response testing, neuropsychological evaluation and as-needed ancillary assessments by psychiatry or physical therapy.^11^ Patients who had dementia, who had language impairments or who were known to have significant discomfort when DBS was turned off were excluded from the study. No patient with orthopaedic complications, such as a past history of digit surgery, who might experience difficulty moving the fingers was recruited. Patients without sufficient postoperative CT imaging for electrode reconstruction were excluded from the study.

### Motor feature selection and processing

We utilized the leap motion controller (LMC; Ultraleap, Mountain View, CA, USA) optical tracking sensor to capture real-time hand location and assess the motor features of bradykinesia. A knowledge of time-variant hand coordinates from the LMC sensor allowed us to quantify hand motion and evaluate the different motor features of bradykinesia independently. LMC sensors have been adopted in previous investigations for objective motor quantification in Parkinson’s disease.^12-14^ Because the change in the amplitude and frequency of movement is most pertinent when patients perform MDS-UPDRS III bradykinesia testing, we chose these two metrics as motor features of interest for subsequent analysis. To best represent changes in the movement of each distal fingertip captured by the LMC sensor, we chose to analyse motor metrics recorded only from the hand movement ‘grasping’ (GR) task administered following the MDS-UPDRS III Section 3.5 ‘Hand Movements’ instructions.

**Figure 1:**
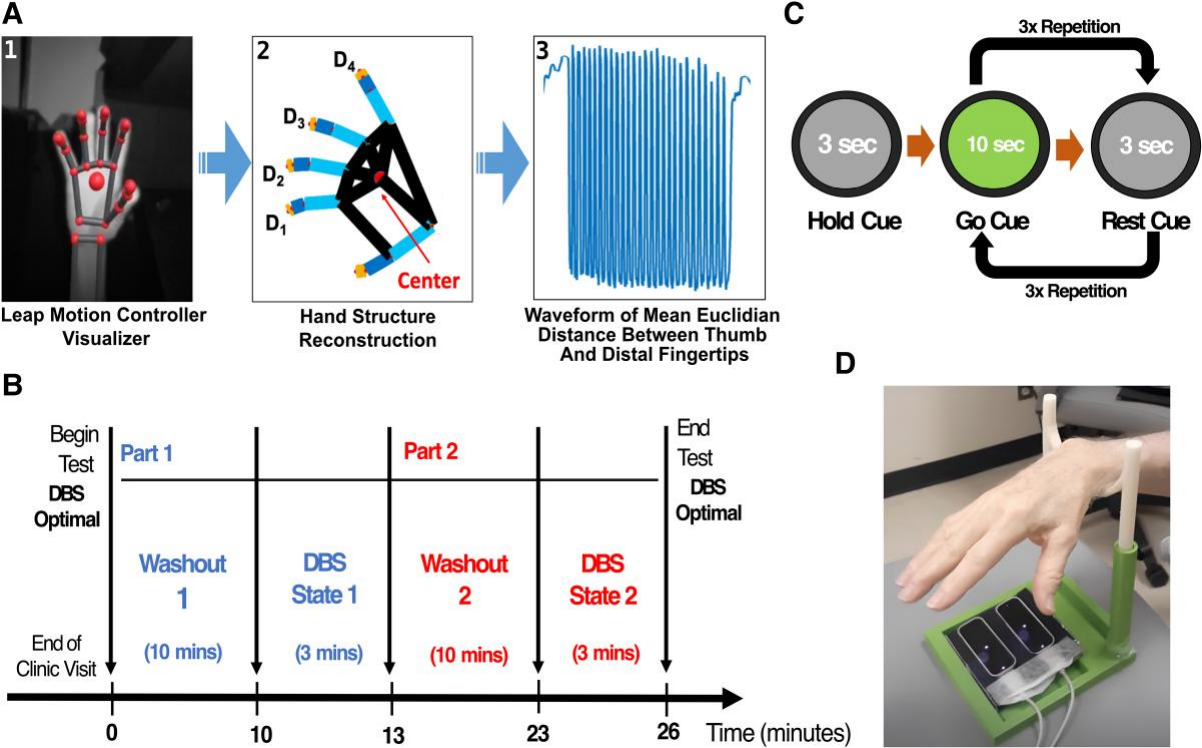
**Study design for motor data collection.** (**A**) Bradykinesia motor features processing flowchart: (1) hand visualization from LMC software user interface, (2) hand coordinate extraction (D1–D4) after LMC recording, (3) the time-variant waveform of hand movement during tasks. (**B** and **C**) Experimental design including wash-in/wash-out periods and randomization of the DBS-ON and DBS-OFF conditions. Before each state, the study participant underwent a 10-min wash-in/wash-out period to remove residual DBS stimulation effects from the preceding state. (**D**) Study apparatus with optical motion sensor and hand rest.

Amplitude and frequency motor metrics were processed and evaluated: the LMC sensor records real-time hand position by tracking individual fingertips and joints (Fig. 1A). From LMC recordings, we were able to reconstruct time-variant coordinates in 3D space of the (i) distal tips of four fingers (D1, D2, D3, and D4) and (ii) centre of the palm while patients were performing the task. Raw movement waveform was captured by taking the mean Euclidian distance between each point of D1, D2, D3, D4, and the centre of the palm for each trial. From this waveform, amplitude and frequency were independently evaluated.

Amplitude was derived as the mean amplitude of movement waveform based on Equation (1a). Frequency was derived as the reciprocal of the mean duration of time taken between each successive peak of the waveform based on Equation (1b), as suggested by Butt *et al*.^12^

Equation (1a). GR task amplitude metric quantification


(1a) $$GR_{\mathrm{amplitude}}=\;\frac{\sum_{k=1}^{4}\;\sqrt{\sum_{n=1}^{3}\;{\left(D_{k,n}-PC_{n}\right)}^{2}}}{4}$$

*D_k_* = distal tip position of the *k*th finger, *C* = palm centre position, n=x,y,zcoordinatesofDk, k=1,2,3,4  (fingernumber).

Equation (1b). GR task frequency metric quantification


(1b) $$GR_{\mathrm{frequency}}=\;\frac{1}{N-1}\overset{N-1}{\underset{i=1}{\sum }}\frac{1}{\left(t_{\mathrm{peak}}(i+1)-t_{\mathrm{peak}}(i)\right)}$$

*N =* total number of peaks, $t_{\mathrm{peak}}$  *=* timepoint at the *i*th peak.

### Study design

Motor data collection using the LMC sensor was performed in two stimulation states: (i) DBS active using clinically effective, stable settings (DBS-ON) and (ii) DBS turned off bilaterally (DBS-OFF), as noted in Fig. 1B. The order of the DBS-ON and DBS-OFF states was randomized such that the rater for motor movements was blinded to the DBS state. While most participants (73.3%) performed under the on-medication condition, others (26.7%) were off-medication or reported unknown medication status. Because there were only 10 min between the 3-min testing sessions, the medication state was very unlikely to change during this brief time period. Patients continued on their normal medication regimen, allowing us to obtain a ‘real-world’ experience of motoric features that respond to acute DBS changes. Participants underwent a 10-min wash-in or wash-out period in each stimulation state before testing to remove the residual stimulation effect from the previous DBS state before undergoing the motor testing. Because prior research suggesting an inverse correlation between disease duration and DBS effect wash-out showed that those with a mean duration of 15 years showed a loss of motor effect after an 8.3-min washout,^15^ we assumed that our cohort with a mean disease duration of 13 years would have a wash-out of the majority of motor signs/symptoms by 10 min.

During the DBS-ON and DBS-OFF states (randomized order), motor testing was conducted to evaluate bradykinesia by asking patients to perform the GR task of the MDS-UPDRS III. For each state, the patients were asked to initially hold the movement for 3 s by looking at the ‘hold’ grey cue on the computer screen, followed by 10 s of ‘go’ green cue and followed by 3 s of ‘rest’ grey cue. The ‘go’ and ‘rest’ cue sequence was repeated three times to collect three trials of motor data per hand (Fig. 1C). The patients were asked to perform movements starting with the right hand, followed by the left hand under each DBS state. Randomization of the DBS-ON and DBS-OFF states was considered a way to control for overall fatigue mounting during the duration of the test or a practice effect.

The severity of bradykinesia for each trial per hand was assessed using the MDS-UPDRS III by a trained, blinded rater. Scores were averaged over the three trials for each stimulation state for each hand. In parallel, we simultaneously quantified amplitude and frequency motor features using the LMC sensor. The LMC sensor was placed directly underneath the palm to maximize the field of view of finger movements (Fig. 1D). A 3D-printed support apparatus was also used to allow study participants to rest their hands during the rest period of the task to maintain the constant height (∼30 cm) between the hand and the sensor.

### Electrode localization

For each patient who underwent LMC motor testing, a preoperative T_1_ MRI and postoperative CT sequence were used for DBS electrode localization and volume of tissue activated (VAT) reconstruction using the Lead-DBS suite.^16^ First, the postoperative CT was coregistered to preoperative T_1_ and T_2_ MRI sequences using the advanced normalization tools (ANTs) + subcortical refine algorithm. The coregistered images then underwent normalization into a common MNI ICBM 2009b Nonlinear Atlas space using the ANTs. Bilateral DBS electrodes were reconstructed in a common atlas space from CT electrode trajectory artefacts using a refined TRAjeCtory and COntact REconstruction (TRAC/CORE) algorithm method.^17^ After DBS electrode reconstruction, the VAT was generated based on activated contacts, stimulation parameters and neighbouring tissue conductivity. The spatial boundary of the binary VAT was defined as regions where the distribution of the electric field was 0.2 V/mm or higher as per previous investigations on neuron modelling.^18-20^

### Mean effect image

A mean effect image (MEI) was produced to display the spatial distribution of the degree of STN-DBS–induced motor feature change. For each patient, the DBS-induced per cent change values (%) of amplitude and frequency of hand movements were assigned to voxels comprising the VAT on the contralateral hemisphere. These ‘numerical’ VATs were then averaged across all patients in the cohort to produce an MEI based on previously published methods.^21,22^ However, due to the limited number of VATs produced from our sample size relative to the hundreds of VATs used in prior investigations, no reliable test statistics could be produced to further threshold the spatial boundaries of MEI that could be used as a patient mask to generate a ‘probabilistic’ stimulation map. Rather, only voxels occupied with at least two VATs were considered when generating MEI. The regions with positive values in the MEI would suggest a cohort-level increase in that motor feature (frequency or amplitude) after DBS is applied and in those with negative values are associated with a decrease in that motor feature.

### Functional connectivity and motor features

To identify cortical and subcortical regions and how polysynaptic activities of these regions are associated with post-DBS amplitude and frequency changes, we performed a correlation analysis between the FC maps. While some theories of DBS’s mechanism focus on monosynaptic connectivity that could be assessed with structural connectivity,^23^ we studied FC in order to assess both monosynaptic and polysynaptic network effects inferred by changes in FC.^24^ FC maps were produced by seeding a normative functional connectome with the VAT of each patient and respective per cent change values (%) of each motor feature. Parkinson’s disease–specific and healthy normative connectomes used were generated based on group-averaged functional MRI signals from 74 Parkinson’s disease patients from Parkinson’s Progression Markers Initiative (PPMI)^25^ and 1000 healthy adults from Brain Genetics Superstruct Project,^26^ respectively.

This approach yielded an ‘R-map’, a whole-brain map that represents the degree of correlation between the FC strength of whole-brain regions with stimulation site and respective changes in motor features. For example, regions in the R-map with positive values note regions where the strength of FC is positively correlated with changes in motor features. This R-map approach was adopted to assess how the degree of functional coactivation of neural activity between the subcortical stimulation site and cortical activity was associated with independent motor feature changes. To better understand which cortical and subcortical region connectivity patterns are significantly associated with motor features, we also performed spatial correlations between motor changes and mean FC intensities within each of the 166 brain regions based on automated anatomical labelling atlas. No regions were selected *a priori*, but all cortical and subcortical masks listed in automated anatomical labelling atlas were studied for unbiased analysis.

### Statistical analysis

Per cent change values (%) of amplitude and frequency were correlated together for each hand using Spearman’s Rho correlation method with statistical significance set at *P* < 0.05. Furthermore, the changes in amplitude and frequency were also correlated with the change in MDS-UPDRS III scores. To address any confounding effect of medication on motor performances, a univariate logistical regression was fit between medication status (on = 1, off = 0) and post-DBS changes in amplitude, frequency, and MDS-UPDRS III. No right and left motor features were significant model parameter estimates for medication status, suggesting a non-significant influence of medication status on motor changes. All graphical and statistical analyses were performed using MATLAB 2021a (MathWorks, Natick, MA, USA) and Prism 9 statistics package (GraphPad Software, San Diego, CA, USA).

## Results

### Demographics and clinical information

**Figure 2:**
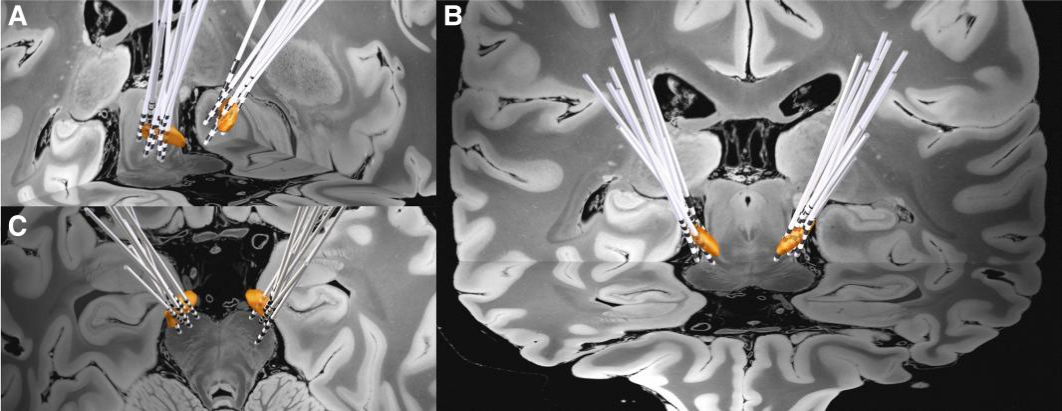
**Grouped STN-DBS electrode localization.** Bilateral DBS electrodes of 15 patients are reconstructed and shown with bilateral STN (3D object) in (**A**) oblique, (**B**) coronal, and (**C**) axial view.

The demographics and clinical information of the study cohort are reported in Table 1. The study cohort consisted of 15 patients with STN-DBS, 13 of whom were males and 2 females. The majority of the cohort (86.7%) were right-handed. Across 15 patients, all preoperative T_1_ MRI [slice thickness (mm) = 1.39 ± 0.08, repetition time (ms) = 1241.43 ± 200.07, echo time (ms) = 3.22 ± 0.20, inversion time (ms) = 635.71 ± 84.25, flip angle (°) = 18.4 ± 3.80] and T_2_ MRI [slice thickness (mm) = 1.87 ± 0.06, repetition time (ms) = 5140.00 ± 492.59, echo time (ms) = 149.24 ± 25.34, flip Aangle (°) = 130.80 ± 8.75] were captured in an isotropic level. All postoperative CT images were captured using a Siemens SOMATOM scanner with 0.75 mm slice thickness. Based on preoperative MRI and postoperative CT sequences, reconstructed DBS electrodes for all 15 patients in the cohort are visualized in Fig. 2.

**Table 1:** Demographics and clinical information

Demographics	n ± SEM (%)
Sex	
Male	13 (86.7%)
Female	2 (13.3%)
Age (years)	70.4 ± 1.6
Age at Parkinson’s disease diagnosis (years)	57.2 ± 2.1
Duration of Parkinson’s disease (years)	13.2 ± 1.2
Handedness	
Right	13 (86.7%)
Left	2 (13.3%)
Medication status^a^	
ON	11 (73.3%)
OFF	3 (20%)
Most affected side	
Right	8 (53.3%)
Left	7 (46.7%)

^a^Medication status of one patient was unknown.

### Association between LMC motor features

**Figure 3:**
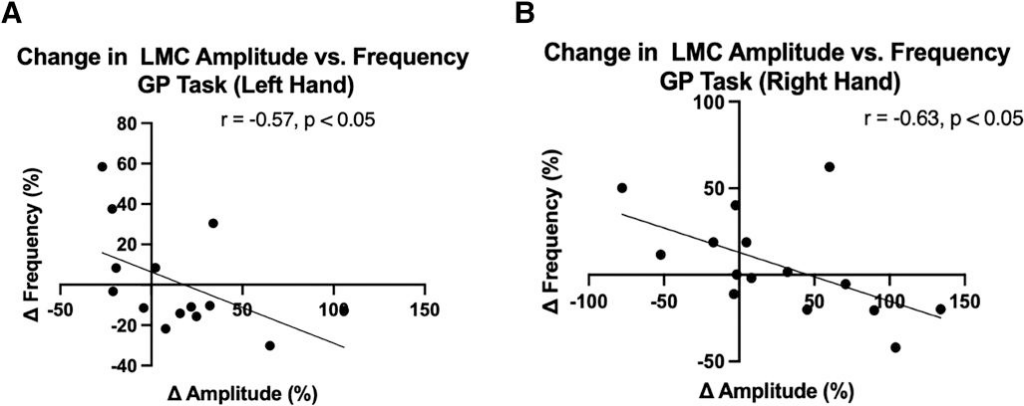
**Association between LMC amplitude and frequency during GR task for (A) left and (B) right hands.** Correlation analysis was performed using Spearman’s rank correlation. Both hands exhibited a negative correlation between the changes in amplitude and frequency after DBS was applied.

Based on the LMC motor features during the GR task, the amplitude and the frequency were independently evaluated. The per cent changes (%) of amplitude and frequency in the DBS-ON versus DBS-OFF states were then correlated against each other as shown in Fig. 3. The change in frequency was negatively correlated with the change in amplitude for both left (*r* = −0.57, *P* = 0.035, Fig. 3A) and right hands (*r* = −0.63, *P* = 0.014, Fig. 3B). To understand the relationship between baseline motor feature performance (e.g. amplitude or frequency deficits during the DBS-OFF period) and DBS-induced change, we looked for correlations between the baseline amplitude and frequency during the GR task and the per cent changes after DBS. The baseline motor performances were not significantly correlated with the degree of DBS-induced changes in frequency of the right (*r* = −0.33, *P* = 0.21) or left hand (*r* = −0.1648, *P* = 0.57) and amplitude of the left hand (*r* = −0.415, *P* = 0.14). However, for right-hand amplitude, post-DBS amplitude changes were strongly correlated with baseline amplitude measures (*r* = −0.675, *P* = 0.0072), suggesting that participants with smaller DBS-OFF amplitudes are more likely to show increased amplitude with DBS activation.

**Figure 4:**
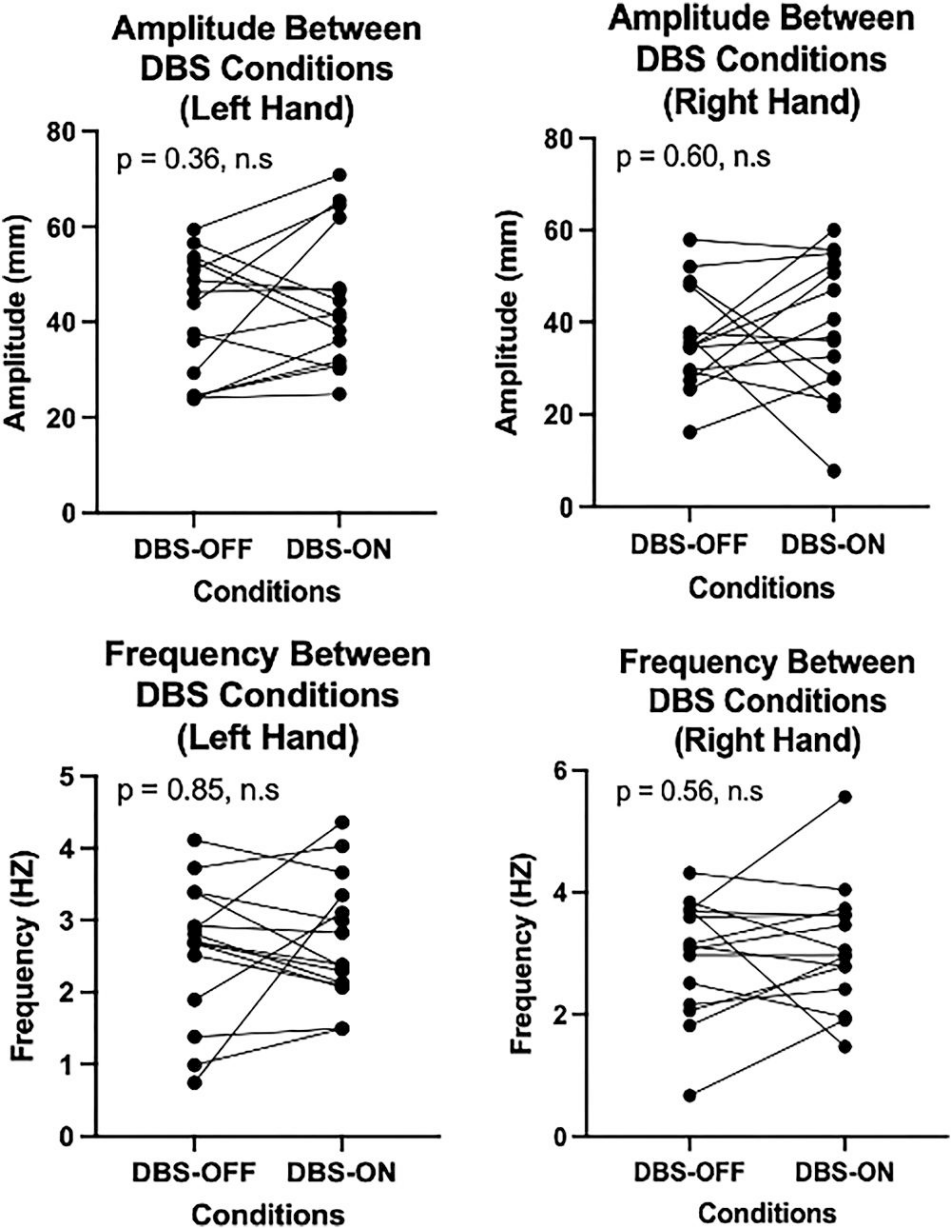
**A comparison of LMC motor feature changes between DBS conditions.** Raw amplitude (*top* row) or frequency (*bottom* row) in the left hand (left column) or right hand (right column) was compared between DBS-OFF and DBS-ON conditions. The Wilcoxon signed-rank test was used for paired analysis across each of the four conditions, resulting in no significant differences in motor features between DBS conditions. Each point in the plot corresponds to the motor performance of each of the 15 patients included in our study.

Additionally, no statistically significant differences in raw motor features between DBS conditions were found (Fig. 4) for the right-hand amplitude (*P* = 0.60), left-hand amplitude (*P* = 0.36), right-hand frequency (*P* = 0.56) and left-hand frequency (*P* = 0.85) based on paired Wilcoxon signed-rank sum test.

### MDS-UPDRS III and LMC motor features

**Figure 5:**
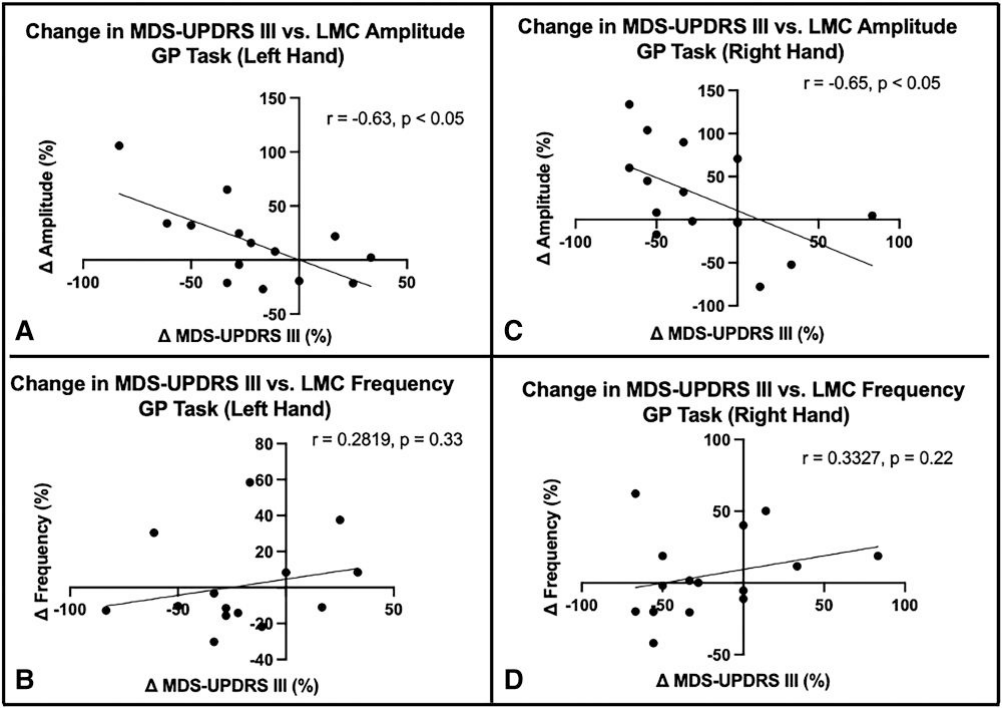
**Association between LMC motor feature changes and MDS-UPDRS III changes during GR task.** The correlation of changes in MDS-UPDRS III with change in amplitude in the left (**A**) and right (**C**) hands and with change in frequency in the left (**B**) and right (**D**) hands. The correlation coefficients are shown for statistically significant correlations.

The strength of correlation was evaluated between changes in MDS-UPDRS III score during the GR task and each of the changes in LMC motor features—amplitude and frequency—caused by DBS activation. The correlation results demonstrate that changes in MDS-UPDRS III features were only significantly correlated with the amplitude changes for both left (*r* = −0.63, *P* = 0.019) and right hands (*r* = −0.65, *P* = 0.01) but not with frequency changes (Fig. 5). This suggests that the assessment of DBS-induced changes in the MDS-UPDRS III hand grasp bradykinesia score is more sensitive to objectively measured changes in amplitude than frequency.

### MEI of LMC motor features

**Figure 6:**
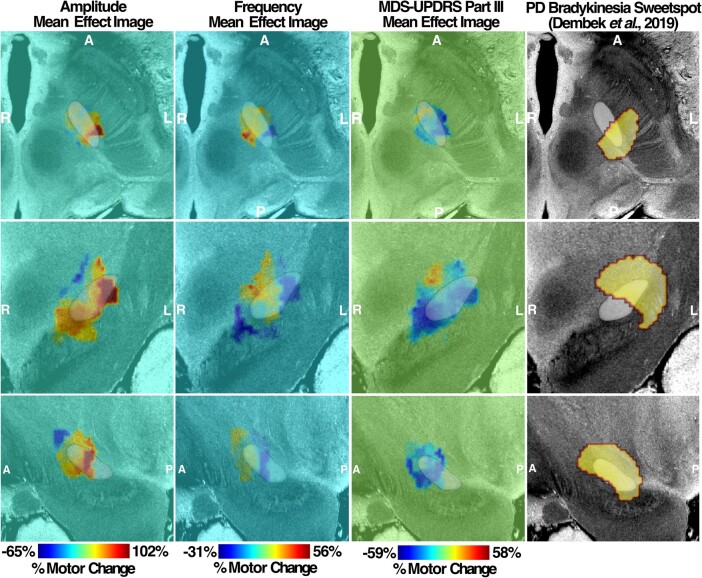
**Left MEI of right-hand LMC motor features.** Amplitude and frequency MEIs are visualized in the first and second columns, respectively, of each stimulation side with overlayed STN (white). The relative warmth or coolness of colouration represents a correlation with an increase or decrease, respectively, in motor features relative to the mean effect. The third column represents MEI of the MDS-UPDRS III changes from our study cohort and the previously published bradykinesia clinical ‘sweetspot’ on the fourth column. A, anterior; P, posterior; R, right; L, left.

**Figure 7:**
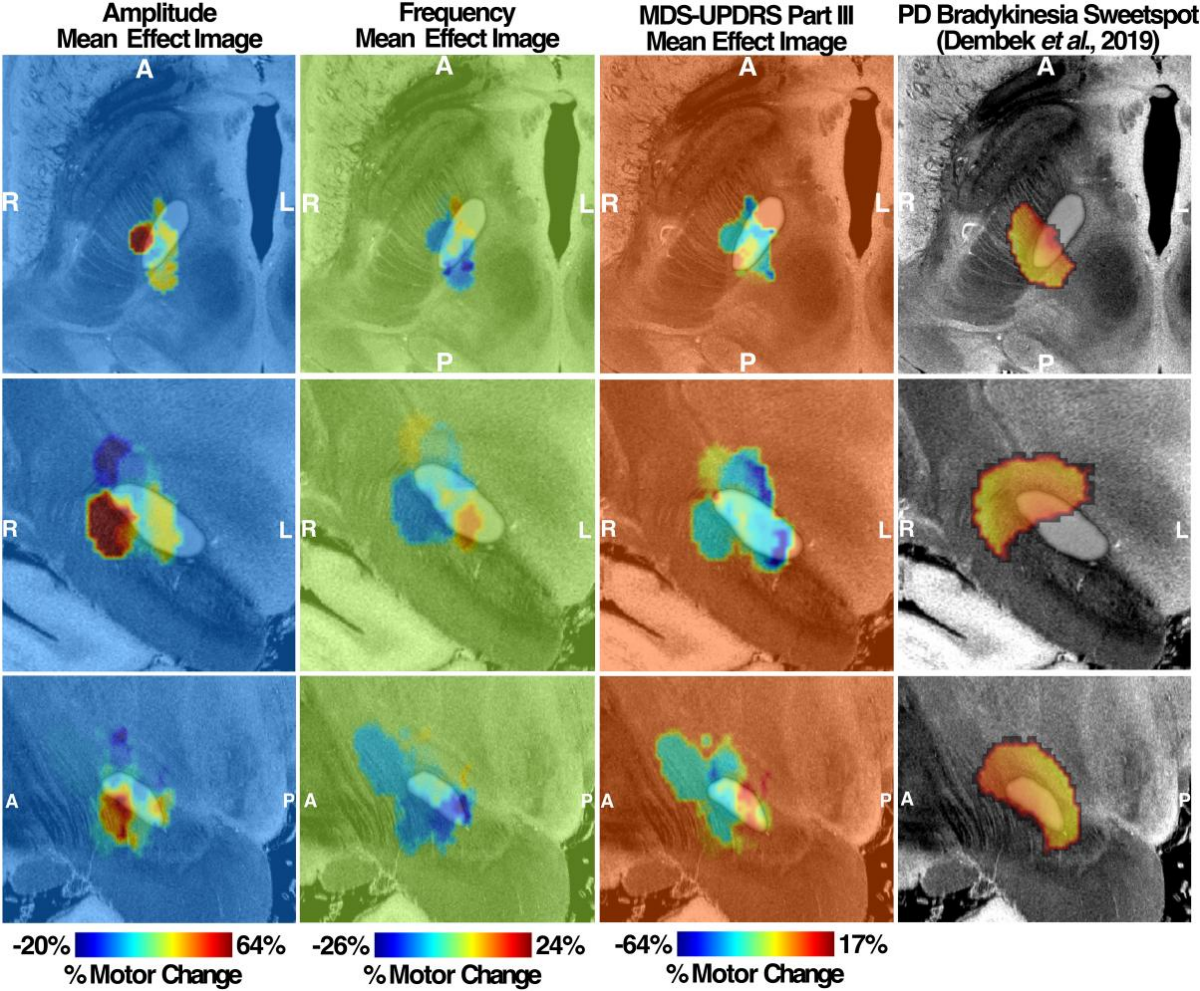
**Right MEI of left-hand LMC motor features.** Amplitude and frequency MEIs are visualized in the first and second columns, respectively, of each stimulation side with overlayed STN (white). The relative warmth or coolness of colouration represents a correlation with an increase or decrease, respectively, in motor features relative to the mean effect. The third column represents MEI of the MDS-UPDRS III changes from our study cohort and the previously published bradykinesia clinical ‘sweetspot’ from the study by Dembek *et al*.^7^ in 2019 on the fourth column. A, anterior; P, posterior; R, right; L, left.

By mapping the amplitude and frequency per cent changes with the VAT for each patient, the MEIs of amplitude and frequency were produced. Based on both amplitude and frequency MEIs, the changes in motor features are topographically represented across the stimulation sites in the STN. The amplitude MEI suggests a gradient of improved DBS-induced movement amplitude moving from anterior-medial regions to posterior-lateral regions for both left STN (Fig. 6) and right STN (Fig. 7). Conversely, stimulation across the same axis was associated with a decrease in frequency. This opposite trend is well reflected based on the spatial inversion of amplitude and frequency MEIs. Furthermore, the direction and the location of MEI of MDS-UPDRS III changes post-DBS matche with that of right and left amplitude MEIs, illustrating a similar stimulation effect in a common STN subregion. The spatial boundary of MDS-UPDRS III MEI also corresponds well to that defined by the Parkinson’s disease bradykinesia ‘sweetspot’ produced from a previous probabilistic sweetspot mapping study.^7^

### R-map of amplitude and frequency changes

**Figure 8:**
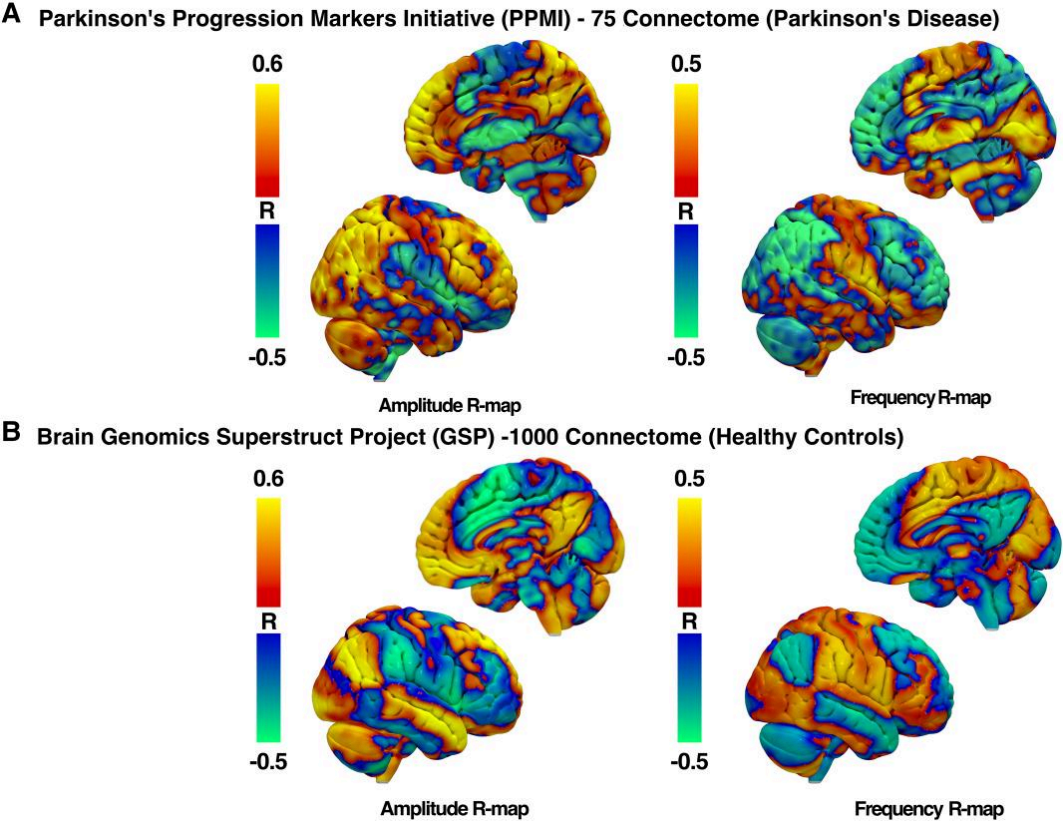
**R-maps of amplitude and frequency changes after DBS.** R-maps were produced from normative functional MRI connectomes from patients with Parkinson’s disease (**A**) and healthy adult controls (**B**). Red–yellow regions represent areas with a positive correlation with changes in FC with the STN-DBS volume of tissue activation and individual motor feature changes. Blue–green regions represent areas with a negative correlation.

We observed a pronounced positive correlation between amplitude changes and FC strengths in prefrontal cortical regions from both Parkinson’s disease–specific PPMI-75 (Fig. 8A) and healthy adult GSP-1000 (Fig. 8B) normative connectomes. Whole-brain R-maps from both connectomes were spatially similar (*r* = 0.3411, *P* < 0.0001) with some regional variability of correlation coefficients. As such, we proceeded with the use of the PPMI-75 normative connectome data because of its disease-specific relevance to our patients who had moderately advanced Parkinson’s disease.

For right-hand GR tasks, larger amplitudes were associated with increased FC with prefrontal motor regions that included, but not limited to, left ventromedial prefrontal cortex (*r* = 0.579, *P* = 0.026), right middle frontal gyrus (*r* = 0.56, *P* = 0.03) and right medial superior frontal gyrus (*r* = 0.678, *P* = 0.007) using the PPMI-75 connectome. When using healthy adult GSP-1000 connectome, correlation coefficients were no longer significant for the left ventromedial prefrontal cortex (*r* = 0.47, *P* = 0.08), right middle frontal gyrus (*r* = 0.31, *P* = 0.25) and right medial superior frontal gyrus (*r* = 0.33, *P* = 0.24).

On the other hand, the R-map for frequency changes was inverted, such that a negative correlation was observed between frequency changes and the strength of FC between the VAT and the prefrontal cortex. The increased frequency of GR was associated with lower FC in the left ventromedial prefrontal cortex (*r* = −0.58, *P* = 0.025), right middle frontal gyrus (*r* = −0.58, *P* = 0.026) and right medial superior frontal gyrus (*r* = −0.57, *P* = 0.029) when using the PPMI-75 connectome. On the other hand, correlation coefficients were no longer significant for the right middle frontal gyrus (*r* = 0.16, *P* = 0.58) and right medial superior frontal gyrus (*r* = −0.49, *P* = 0.06) but preserved for the left ventromedial prefrontal cortex (*r* = −0.56, *P* = 0.034).

## Discussion

In this study, we have investigated the distinctive effect of STN-DBS on components of bradykinesia—amplitude and frequency—in patients with Parkinson’s disease. With the activation of clinically effective STN-DBS, the changes in amplitude were negatively correlated with those in frequency or vice versa. Furthermore, the DBS stimulation sites associated with an increase in amplitude were spatially distinct from those with an increase in frequency in the STN. This inversion of neural substrates representing amplitude and frequency changes persisted from the subcortical basal ganglia level to the global cortical level through whole-brain connectivity analysis. To the best of our knowledge, this study is the first to report the anatomical segregation of the STN motor subregions that differentially encode amplitude and frequency changes post-DBS and on how this segregation was maintained across FC patterns at the cortical level.

We have closely considered the relationship between amplitude and frequency in the GR task and have demonstrated a significant negative correlation between amplitude and frequency changes observed in both hands (*r* = −0.57 for left hand, *r* = −0.63 for right hand) after DBS; as amplitude increases, the mean frequency slows. If velocity is relatively constant, this trade-off would be inherent to increasing amplitude. However, this negative correlation is not solely explained by this trade-off with reduced frequency as amplitude increases since there were stimulation settings that improved frequency at the cost of amplitude, relative to DBS being OFF (Fig. 3). Interestingly, the association between these motor features and the MDS-UPDRS III bradykinesia rating further revealed that the changes in bradykinesia were significantly driven by changes in amplitude, rather than frequency. Specifically, the increase in amplitude was correlated with the improvement of bradykinesia scores for both hands despite the slowing of hand GR with larger amplitudes. While we found that no previous studies have explored the direct effect of DBS on bradykinesia components, one study reported that MDS-UPDRS III was also negatively correlated with amplitude during GR task using a wearable kinematics device in the baseline condition.^27^

After the changes in motor features were mapped to the volumes of stimulated tissue across all subjects to create the MEI, the MEI valence (‘sweet spot’ versus ‘sour spot’) was inverted between amplitude and frequency changes: stimulation location with the ‘maximum increment’ of amplitude corresponded to that with the ‘maximum decrement’ of frequency. The ‘sweet spot’ for amplitude changes was more posterior dorsolateral and for frequency, more anterior medial in the STN. One could hypothesize that ‘effective’ STN stimulation is increasing amplitude but decreasing frequency and that the frequency ‘sweet spot’ is just the absence of a robust amplitude benefit. However, participants with the stimulation of this region actually show improved frequency over DBS-OFF, at the cost of amplitude (smaller, faster movements compared with DBS-OFF). Furthermore, our results suggest that except for amplitude measures on the right hand, DBS-OFF motor measures had no significant impact on overall changes in frequency or amplitude after DBS is applied, highlighting that the observed motor changes are specific to DBS and the location of the STN stimulated. A negative correlation result between DBS-OFF right-hand amplitude measurement and its change after DBS is turned on further illustrates that individuals with smaller DBS-OFF amplitude are more likely to show a substantial increase in amplitude after DBS is applied.

Based on this spatially graded distribution within STN, we postulate that the negative correlation between amplitude and frequency changes was driven by the inversive representation within the shared neural substrate and the network connectivity of these two different STN subregions. R-maps produced from PPMI-75 connectome between amplitude and frequency were spatially inverted, with regions with significant correlations in the middle frontal gyrus, medial superior frontal gyrus and ventromedial prefrontal cortex. While the strength and directionality of the correlation were reduced in R-maps produced from the GSP-1000 healthy control connectome, it is thought that disease-specific connectome may better capture the network-level behaviours specific to and particularly affected in patients with Parkinson’s disease than connectomes generated from healthy controls.^28^ Therefore, it is feasible that the correlation results from anatomical regions identified from the PPMI-75 connectome may provide stronger evidence of neural substrates involved in both motor control and affected in patients with Parkinson’s disease.

In a sequential movement such as the GR task, the pre-supplementary motor area (pre-SMA), part of the medial superior frontal gyrus and medial frontal cortex, has been suggested to be involved in the initiation of self-generated action sequences.^29-32^ During a self-initiated movement task, the FC between STN and pre-SMA region has been reported to be strengthened for both healthy patients and patients with Parkinson’s disease by employing the hyper-direct and indirect basal ganglia pathways.^33^ STN-DBS may interfere in the STN–pre-SMA connection to modulate performances in movement initiation in GR, which may emerge as alterations in the motor frequency and amplitude from baseline condition. The magnitude and the direction changes of two motor features (i.e. increment or decrement) after STN-DBS may be determined based on the locations of individual VATs relative to ‘amplitude hotspots’ or ‘frequency hotspots’ spatially distributed across the dorsolateral–ventromedial axis of the STN. However, the precise STN–pre-SMA connections associated with amplitude and frequency changes are yet unknown and would warrant further investigations.

The spatial distribution of DBS’s differential effect on the amplitude of rapid movements is consistent with the existing literature. That targeting dorsolateral STN for DBS was associated with the greatest bradykinesia improvement^7,34^ is consistent with our finding, such that posterior dorsolateral STN resulted in the greatest improvement of amplitude changes and the greatest improvement in MDS-UPDRS III bradykinesia rating. The dorsolateral region of STN has further been shown to share strong structural connectivity with sensorimotor cortical areas such as the primary motor cortex area and SMA. Conversely, the more medial region of STN encompassing the ‘sweet spot’ for frequency increment is spatially proximal to the associative subregion of STN that shares strong connectivity with the associative areas of prefrontal cortices.^35,36^ The differential responses in amplitude and frequency changes thus may be attributed to the activation of separate STN prefrontal networks from DBS. Expanding on the network-level stimulation effect on motor features, we have performed an R-map analysis correlating the degree of FC seeding VATs and changes in motor features.

Several limitations exist in this study. This is a preliminary study performed across a limited cohort of 15, and future studies with a larger sample size are strongly encouraged to replicate our findings. With a limited number of patients and VATs of 15, we generated the raw MEI but could not take additional steps to impose spatial constraints to further generate a ‘significant’ stimulation map as done in previous investigations. A subsequent study with a larger patient cohort is required to further validate and demarcate the precise spatial boundaries of frequency and amplitude ‘sweet spots’. Furthermore, in addition to amplitude or frequency, the MDS-UPDRS III rating is composed of other motor features, including hesitations or temporal decrement. We chose to focus on amplitude and frequency because of their canonical influence on shaping the bradykinesia rating and to clear pre-processing steps to evaluate them from raw motor data. Future studies should aim at how performances in self-generated action sequences as GR task are reflected and altered across multiple motor features of bradykinesia after STN-DBS. We do not have a data-driven explanation for the observation that baseline ‘off stimulation’ amplitude correlated inversely with the stimulation-induced change in amplitude only on the right side but not the left. One potential explanation could be that because 87% of the cohort was right handed, greater effort was placed on programming the right hand, and thus a larger degree of improvement drove a stronger correlation (that also trended in the same direction in the left hand).

Lastly, we focused on the isolated effect of STN-DBS and aimed to run our 26-min paradigm during whatever medication state in which they presented, hoping to look at the subtle changes in bradykinesia detected with the LMC even in the fully or partially medicated state. It is possible that medication status changed during our paradigm. Nevertheless, because the paradigm was relatively short (10 min between two 3-min testing periods), we are confident that both DBS-ON and DBS-OFF test periods were performed in the same state of medication effect. We randomized the stimulation state (‘DBS-ON’ or ‘DBS-OFF’) so that if a medication wearing-off or kick-in effect were to occur, it would not systematically be linked with the stimulation ‘on’ or ‘off’ state. In addition, depending on individual variability, it must be noted that a 10-min wash-out between each DBS condition may not be sufficient, and that relative changes in motor measures and MDS-UPDRS III scores may be attenuated and cause a Type II statistical error. Nonetheless, even with a potential attenuation of the degree of motor changes, we still captured the differential association between amplitude and frequency changes driven by DBS and its stimulation location within the STN, suggesting that the finding is robust against a limited sample size.

## Conclusion

In this study, we demonstrated a differential effect of STN-DBS on bradykinesia motor features (amplitude and frequency), an effect that also translates to differential network engagement. Improvements in hand movement amplitude were negatively correlated with movement frequency and were associated with the stimulation of a different STN subregion than that which was associated with an improvement in frequency and a decline in amplitude. The degree of motor changes was represented as a spatial gradient across the ventromedial–dorsolateral axis of the STN. FC analysis between stimulated volumes in STN and cortical areas showed a differential pattern for improvement in amplitude versus frequency of movements.

## Data Availability

The data that support the findings of this study are available from the corresponding author upon reasonable request.
